# TMPRSS2-ERG fusions confer efficacy of enzalutamide in an in vivo bone tumor growth model

**DOI:** 10.1186/s12885-019-6185-0

**Published:** 2019-10-21

**Authors:** Louie Semaan, Navneet Mander, Michael L. Cher, Sreenivasa R. Chinni

**Affiliations:** 1 Department of Urology, Wayne State University School of Medicine, 9245 Scott Hall, 540 E. Canfield Avenue, Detroit, MI 48201 USA; 2 Department of Pathology, Wayne State University School of Medicine, Detroit, MI 48201 USA; 3 Department of Oncology, Wayne State University School of Medicine, Detroit, MI 48201 USA

**Keywords:** TMPRSS2-ERG, Castrate resistant prostate cancer, Enzalutamide, Bone tumors, Androgen receptor, Androgen biosynthetic enzymes, AKR1C3, HSD3B1 and HSD3B2

## Abstract

**Background:**

Castrate Resistant Prostate Cancer (CRPC) is an advanced disease resistant to systemic traditional medical or surgical castration, and resistance is primarily attributed to reactivation of AR through multiple mechanisms. TMPRSS2-ERG fusions have been shown to regulate AR signaling, interfere with pro-differentiation functions, and mediate oncogenic signaling. We have recently shown that ERG regulates intra-tumoral androgen synthesis and thereby facilitates AR function in prostate cancer cells. We hypothesize that enzalutamide treatment will be more effective in cells/tumors with TMPRSS2-ERG translocations because these tumors have increased AR signaling.

**Methods:**

ERG knockdown was performed with VCaP cells using lentiviral infections to generate VCaP ERGshRNA cells and control VCaP scr cells with scrambled shRNA. Cell-growth analysis was performed to determine the effect of enzalutamide. Reverse transcription, quantitative real-time PCR (RT-qPCR) was used to determine the expression of AR responsive genes. Luciferase tagged VCaP scr and shRNA infected cells were used in an intra-tibial animal model for bone tumor growth analysis and enzalutamide treatment used to inhibit AR signaling in bone tumors. Western blotting analyzed VCaP bone tumor samples for ERG, AR, AKR1C3 and HSD3B1 and HSD3B2 expression.

**Results:**

Enzalutamide inhibited the growth of VCaP scr cells more effectively than shERG cells. Analysis of AR responsive genes shows that Enzalutamide treatment at 5 micromolar concentration inhibited by 85–90% in VCaP Scr cells whereas these genes were inhibited to a lesser extent in VCaP shERG cells. Enzalutamide treatment resulted in severe growth inhibition in VCaP scr shRNA cells compared to VCaP shERG cells. In bone tumor growth experiment, VCaP ERG shRNA cells grew at slower than VCaP scr shRNA cells. Androgen biosynthetic enzyme expression is lower VCaP shERG bone tumors compared to VCaP scr shRNA bone tumors and enzalutamide inhibited the enzyme expression in both types of tumors.

**Conclusions:**

These data suggest that ERG transcription factor regulates androgen biosynthetic enzyme expression that enzalutamide treatment is more effective against VCaP bone tumors with an intact ERG expression, and that knocking down ERG in VCaP cells leads to a lesser response to enzalutamide therapy. Thus, ERG expression status in tumors could help stratify patients for enzalutamide therapy.

## Background

Androgen deprivation therapies in prostate cancer patients are effective in reducing systemic levels of androgens. However, studies demonstrate that intratumoral androgens do not change significantly, and those levels are sufficient to activate AR growth signaling pathways [1–4], suggesting that, in a castrate environment, tumors have the capability to make local androgens. Subsequent studies showed that in CRPC, tumor tissues exhibit enhanced levels of androgen biosynthetic enzyme expression [5, 6]. Production of tumoral androgens in the castrate environment can be enhanced from adrenal precursors such as dehydroepiandrosterone (DHEA) and androstenediol, and directly from cholesterol. Several studies suggest that, downstream of the CYP17 gene in the androgen pathway, there could be a viable alternate target for intratumoral androgen production [7–10]. How expression of these androgen biosynthetic enzymes is regulated in CRPC remains to be investigated. We showed for the first time that ERG transcription factor regulates androgen biosynthesis and subsequent AR activation in prostate cancer cells [11]. ERG transcription factor is expressed in PC tumors from TMPRSS2-ERG fusion gene and the presence of this fusion is highly prevalent in PC patients, including CRPC. The presence of TMPRSS2-ERG fusions is associated with high grade disease [12], and different subsets of rearrangements including 2 + Edel, T2-E4, and the presence of a 72 bp insert in the ERG gene are associated with aggressive disease characteristics [13–15]. ERG has been shown to bind AR responsive gene promoters throughout the genome and regulate AR signaling, interfere with prodifferentiation functions, and mediate oncogenic signaling [16]. Also cooperation between AR and ERG drives invasive adenocarcinoma [17] even in the castrate environment [18]. However, mechanisms underlying the cooperation of AR and ERG with each other in a low androgen environment remain unknown. In a low androgen CRPC state, AR reprograms gene expression for enhanced cell cycle progression [19], which is distinct from the prodifferentiation function. ERG fusion status is also related to abiraterone acetate therapy responsiveness, where fusion positive patients responded well to therapy, suggesting a role of the fusion gene in androgen production [20]. Our previous study supports this notion and defined a feed forward loop consisting of TMPRSS2-ERG fusions, androgen biosynthetic enzymes and AR activation operating in prostate cancer cells which drives intracellular DHT production. In this study, we tested the biological significance of ERG induced androgen production on bone tumor growth and targeting AR signaling with enzalutamide.

Bone metastases are highly prevalent occurrences in both hormone-naïve and CRPC patients. Androgen receptor expression is prevalent in bone metastases where both full length [21] and shorter AR variants [22, 23] are expressed in metastasized prostate tumor cells. Nuclear AR staining in bone metastases associate with poor outcome in patients, suggesting AR signaling in promoting bone tumor growth [21]. At a cellular level, AR expression and signaling has been active in mature osteoblasts, osteocytes, and stromal cells [24–26] that maintain normal bone function. In support of this argument, a clinical trial with abiraterone acetate showed that treated patients have lower testosterone levels in bone marrow tumor biopsies [27], suggesting the prevalence of androgen production in bone metastatic sites.

Our previous study demonstrates that AR/ERG/Androgen biosynthetic enzymes form a feed forward loop in TMPRSS2-ERG fusion positive cells for androgen production and growth [11]. This study is focused on determining the impact of breaking this feedforward loop on bone tumor growth. We utilized ERG knockdown approach in TMPRSS2-ERG positive cells and targeting the AR function with enzalutamide for bone tumor growth. We show that ERG positive cells/tumors are highly responsive to enzaluatimde treatment. AR responsive genes in CRPC state are inhibited by enzaluatamide treatment in ERG positive cells. Androgen biosynthetic enzyme gene expression is inhibited in ERG knockdown tumors andenzalutamide significantly inhibited androgen biosynthetic enzyme expression in ERG positive tumors likely through abrogating the feedback back mechanism involving AR/ERG/androgen biosynthetic enzyme expression [11]. This data supports the notion that TMPRSS2-ERG fusion positive tumors are better responders to enzalutamide therapy.

## Methods

### Cell lines and treatments

The parental VCaP cell line was obtained from ATCC (Catalogue number CRL-2876) and cultured in DMEM media. Preparation of VCaP Scrambled shRNA and VCaP shERG RNA cells were described previously [11]. Cell lines were cultured in a humidified incubator with 5% CO_2_ at 37 °C. All media were supplemented with 2 mM glutamine, 100 units/mL penicillin, and 100μg/mL streptomycin (Life Technologies Inc., Carlsbad, CA). VCaP Scrambled shRNA Control and VCaP shERG RNA cells were maintained in ATCC-DMEM media supplemented with (0.3μg/mL) puromycin. Both cell lines were authenticated with STR analysis (Genomics core at Michigan State University, East Lansing, MI) and shown to have markers respective for each cell line as established by ATCC and also tested for mycoplasma contamination prior to use, with Venor-GeM mycoplasma detection kit (Sigma Biochemicals, St. Louis, MO).

### Cell proliferation

VCaP Scrambled shRNA and VCaP ERG shRNA cells were seeded in a Cell-Bind 96-well, black culture plate at a cell density of 10,000 cells per well in 100 μL media supplemented with 10 μM of HEPES. Cells were seeded in quadruplicates and incubated for 24 h (to allow adhesion). The following day cells were treated with 5, 10 and 20 μM of enzalutamide. Cells were assessed for growth at the indicated times (2, 3, and 5 days) by CyQuant NF cell proliferation fluorescence assay kit (Cat# C35006- Invitrogen Molecular Probes). Growth media and enzalutamide treatment were replenished at day 3 for continued drug effect. Relative fluorescence units were quantified using a fluorescent plate reader.

### Gene expression

VCaP Scrambled shRNA and VCaP ERG shRNA cells were seeded in 6 well plates at a cell density of 5.0 × 10^5^ cells per well. Cells were treated with 5 and 20 μM of enzalutamide for 5 days, with media replenished on day 3. On day 5, RNA was extracted using the Trizol (Invitrogen, Carlsbad, CA) method. For RT-qPCR experiments, cDNA was synthesized from total RNA using the iScript cDNA synthesis kit obtained from Bio-Rad (cat.# 170–8891) with an Eppendorf Mastercycler. For RT-qPCR experiments cDNA was amplified with primers and SYBR green from Maxima SYBR Green/ROX qPCR Master Mix (2X) obtained from Thermo Scientific (cat.# KERK0221) using an Eppendorf Mastercycler Realplex^2^ qPCR machine. Fold change levels among gene expression were quantitated using comparative Ct method. In this study AR responsive genes related to differentiation such as PSA and TMPRSS2, and CRPC genes, such as, UBE2C, CDK1, CCNA2 and CDC20 were analyzed.

PCR primers are listed in Table 1.

**Table 1:** **Table 1**

CRPC Genes	Forward (5′ ➔ 3′)	Reverse (5′ ➔ 3′)
UBE2C	TGGTCTGCCCTGTATGATGT	AAAAGCTGTGGGGTTTTTCC
CDK1	CCTAGTACTGCAATTCGGGAAATT	CCTGGAATCCTGCATAAGCAC
CCNA2	CAGAAAACCATTGGTCCCTC	CACTCACTGGCTTTTCATCTTC
CDC20	CCTCTGGTCTCCCCATTAC	ATGTGTGACCTTTGAGTTCAG
TMPRSS2	CAGGAGTGTACGGGAATGTGATGGT	GATTAGCCGTCTGCCCTCATTTGT
GAPDH	ATCACCATCTTCCAGGAGCGA	GCCAGTGAGCTTCCCGTTCA

### In vivo studies

For in-vivo studies VCaP Scrambled shRNA Control and VCaP ERG shRNA cells were infected with Luciferase-2 lentiviral particles in order to capture in-vivo bioluminescence images of tumors. Castrated five-week old male C.B.-17 severe combine immunodeficient (SCID) mice (Taconic Farms, Germantown, NY) were used in this study. All the procedures including animal housing, surgery, imaging, the methods of anesthesia and euthanasia prior to tumor tissue analysis were performed as per the institutional animal care and use committee approved protocol. For intratibial implantation, 1.0 × 10^6^ cells in a 10 μl volume were injected per bone. Animals were imaged periodically to measure bone tumors. Animals with established bone tumors were randomized and treated with a dose of 50 mg/kg body weight of enzalutamide or placebo control by oral gavage. Luciferase imaging of tumors were performed with Carestream Invivo Xtreme system. At the end of the experiment animals were euthanized with carbon dioxide inhalation method and bone tumors were collected for Western blot analysis and quantifying tumoral androgen levels.

### Western blot analysis

VCaP shscrambled and shERG bone tumor were weighed and supplemented with (100 mg tumor in 900uL RIPA^++^) RIPA buffer (50 mM Tris-HCl, pH 8.0, 150 mM NaCl, 0.5% sodium deoxycholate, 1% NP-40) plus protease (1X complete Mini protease inhibitor cocktail tablets) and phosphatase (10 mM sodium pyrophosphate, 25 mM β-glycerophosphate, 25 mM NaF, 2 mM NaVO_3_) inhibitors (RIPA^++^) and homogenized using a Precellys 24 Homogenizer (PEQLAB, Wilmington, DE), mixing end-over-end at 4 °C for 15 min followed by centrifugation at 15,000×g for 15 min. Protein concentrations were determined using BCA reagent (Pierce), and 30-40 μg protein was analyzed by Western blot analysis. Membranes were blocked with 5% BSA/TBST and probed with primary antibody in 5% BSA/TBST. Membranes were probed with HRP-linked secondary antibody in 5% BSA/TBST. Protein bands were detected using enhanced chemiluminescence substrate and autoradiography film. Primary and secondary antibodies are listed in supplementary table below. Densitometry was performed using ImageJ software (NIH). The details of antibodies used in Western blot analysis were provided in Table 2.

**Table 2:** **Table 2**

Primary Antibody	Vendor	Catalog number	Dilution
ERG 1/2/3	Santa Cruz Biotechnology	sc-28,680	1:5000
AR (N-20)	Santa Cruz Biotech	sc-816	1:1000
GAPDH	Santa Cruz Biotech	sc-25,778	1:10000
AKR1C3	Sigma Aldrich	A6229	1:1000
HSD3B1	Sigma Aldrich	WH0003283M1	1:1000
HSD3B2	Sigma Aldrich	SAB1402232	1:1000
Secondary Antibody	Vendor	Catalog number	Dilution
Anti-rabbit IgG, HRP	Cell Signaling Technology	7074S	1:5000
Anti-mouse IgG, HRP	Cell Signaling Technology	7076S	1:5000

### Tumoral androgen quantitation

For tumoral testosterone measurements, total lipids were extracted from bone tumor using a Sep-pak cartridge obtained from Waters Corporation (Milford, MA, WAT023590). Lipid samples were eluted with acetonitrile into HPLC/MS/MS vials obtained from MicroSOLV Technology (Eatontown, NJ, 9512S-0CV vials, 9502S-10C-B tops) and subjected to HPLC/MS/MS analysis at the Wayne State University Lipidomics Core Facility as described previously [11].

### Statistical analysis

Statistical analyses were performed using GraphPad Prism Version 5.0.

## Results

### TMPRSS2-ERG fusion positive cell growth is sensitive to enzalutamide exposure

**Fig. 1:**
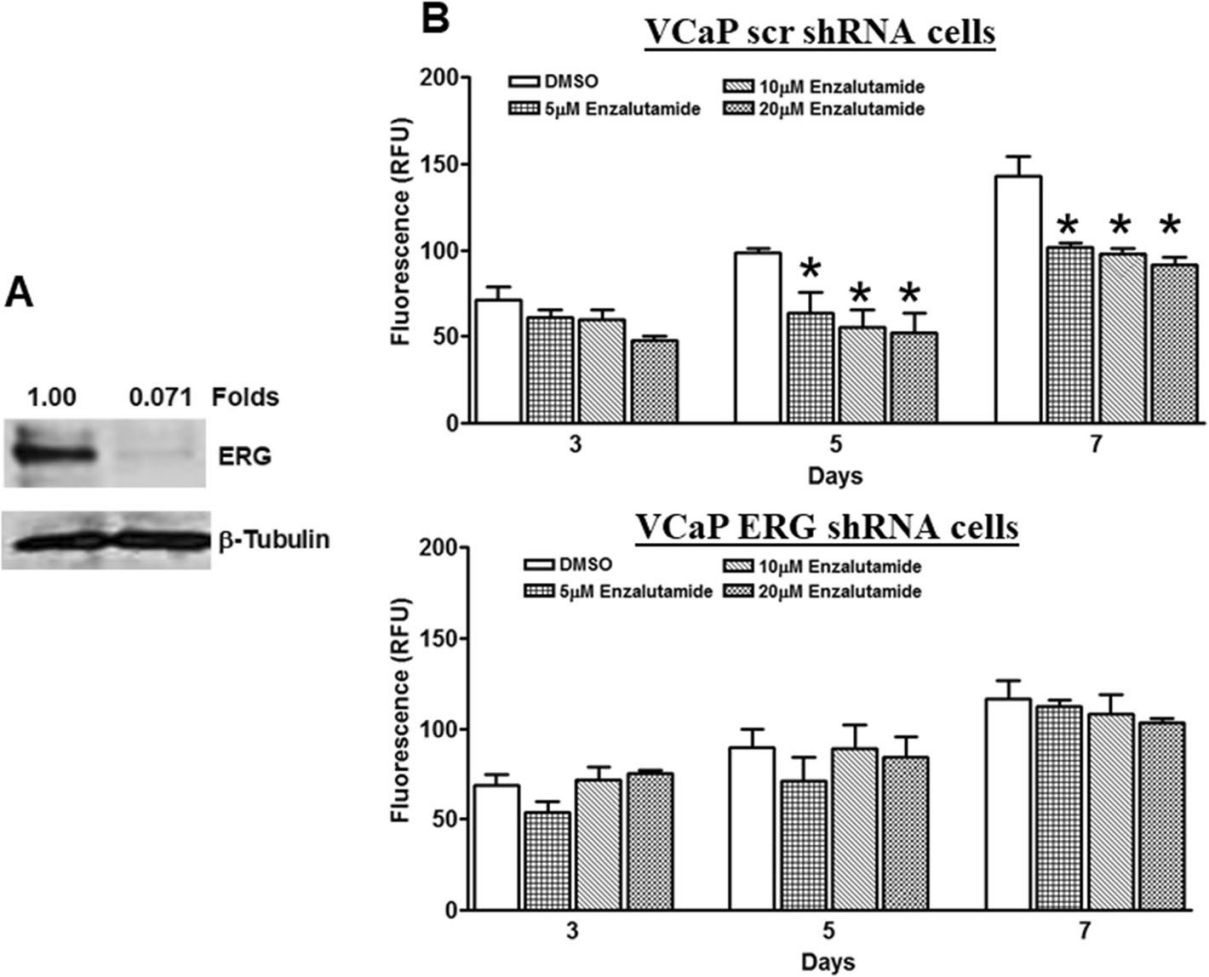
Enzalutamide inhibits TMPRSS2-ERG fusion positive cancer cell growth. **a** Western blot analysis of VCaP Scr shRNA and VCaP ERG shRNA cells for ERG and b-tubulin expression. ERG expression was normalized for b-tubulin and fold differences between VCaP Scr shRNA and VCaP ERG shRNA cells were shown. **b** Cell growth analysis of VCaP scr shRNA and VCaP ERG shRNA cells treated with 5–20 μM concentrations of enzalutamide for 3, 5 and 7 days. * Indicate the *p* < 0.05 between groups (DMSO vs. enzalutamide treatment) using student t-test and *n* = 3

Our previous study showed that ERG factor regulates androgen biosynthesis and that intracellular androgen production activates AR responsive gene expression [11]. Here we tested whether ERG factor could be a better marker for androgen receptor mediated cell growth. We have previously showed that VCaP cells in culture are known to produce androgens and knockdown ERG with shRNA leads to decrease in androgen production [11]. We generated a pair of isogenic VCaP cell lines, where we stably infected with ERG shRNA to knock down ERG expression, or with scrambled shRNA as a control cell line with intact ERG expression, Western blot analysis show that ERG factor expression is significantly down regulated in VCaP ERG shRNA cells compared to VCaP scr shRNA cells (Fig. 1a). Treatment with enzalutamide showed that VCaP scr shRNA cells are sensitive to cell growth inhibition starting from 5 μM concentration at day 5 and 7; whereas, ERG knockdown cells did not show cell growth inhibition at these concentration (Fig. 1b). Together these data suggest that ERG expression confer the responsiveness to enzalutamide treatment.

### Enzalutamide inhibits AR dependent gene expression in ERG positive cells

**Fig. 2:**
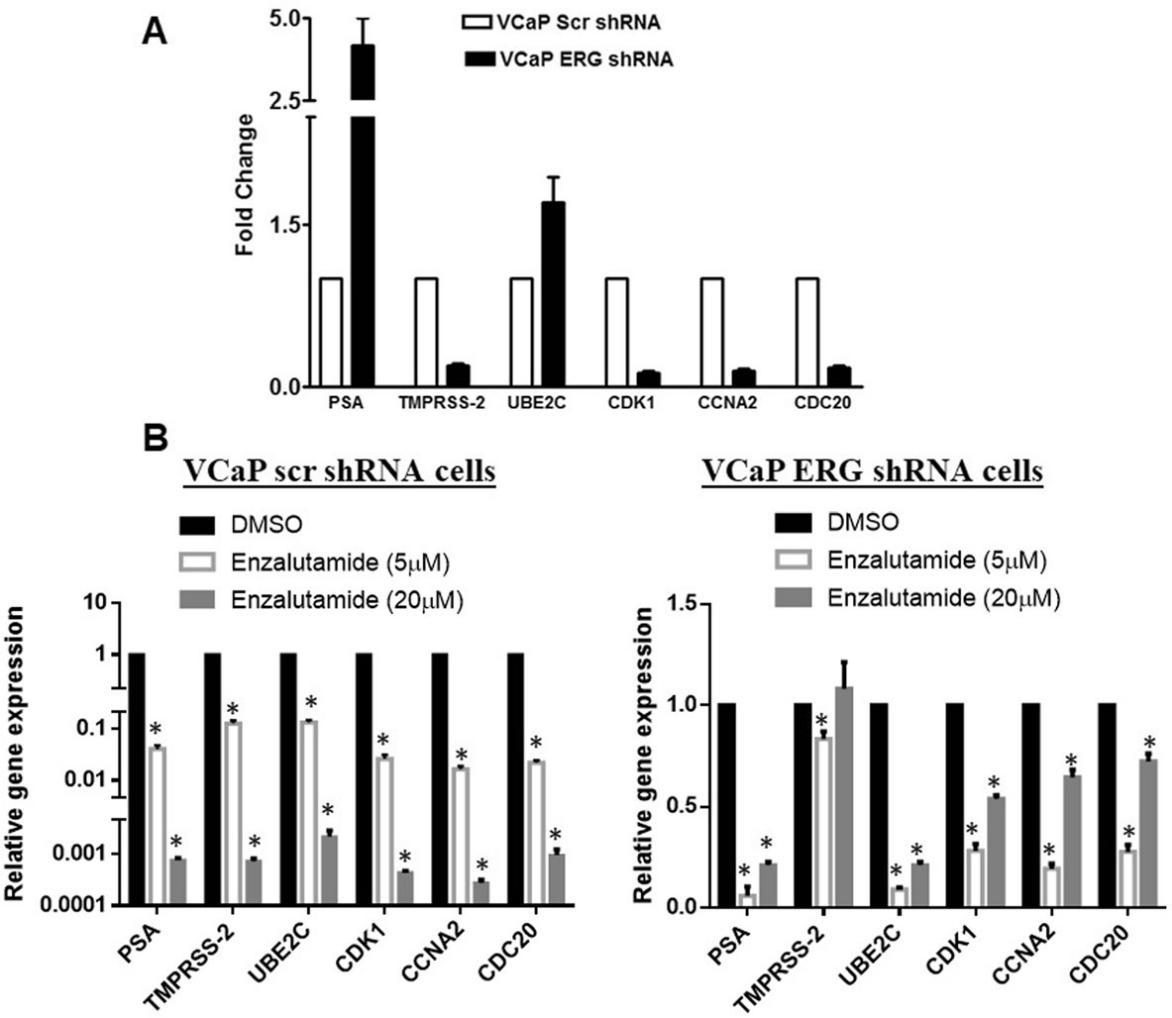
AR responsive gene expression in TMPRSS2-ERG fusion positive cells and enzalutamide regulation of AR responsive genes. QPCR analysis of AR responsive genes in VCaP scr shRNA and VCaP ERG shRNA cells (**a**) and changes in AR responsive genes upon enzalutamide treatment (**b**). * Represents *p* < 0.001 using student t-test and n = 3

Previous studies defined AR gene signatures specific to the CRPC phase of disease are selectively upregulated M-phase cell cycle genes [19], and expression of these genes in ERG intact and knockdown cells were evaluated. Data showed that CDK1, CCNA2 and CDC20 are downregulated in ERG knockdown cells, whereas UBE2C is upregulated in ERG knockdown cells; similarly, among AR responsive genes, TMPRSS2 is downregulated in ERG knockdown cells and PSA is upregulated in ERG knockdown cells (Fig. 2a). ERG has been shown to regulate several AR responsive genes in PC cells [16] and its knockdown in VCaP cells resulted in upregulation of these genes.

Both VCaP scrambled shRNA (high ERG) and VCaP ERGshRNA (low ERG) cells were exposed to DMSO (as a control) or 5 and 20 μM enzalutamide for 5 days. QPCR analysis of AR responsive genes TMPRSS2 and PSA, and CRPC genes UBE2C, CDK1, CCNA2 and CDC20 shows that expression of all these genes was highly downregulated at both concentrations in VCaP scrambled shRNA (high ERG) cells (Fig. 2b). At 5 μM concentration of enzalutamide, 85–95% of these gene expressions were downregulated and at 20 μM concentration these gene expressions were downregulated to the extent of 3 log units in ERG intact cells. However, in VCaP ERG shRNA (low ERG) cells, enzalutamide did not inhibit the TMPRSS2 gene expression at 5 μM concentration and all other genes were inhibited to a lower extent compared to ERG intact cells, whereas at 20 μM concentration the extent of inhibition in all genes is less than in high ERG cells. Together, these data suggest that AR inhibition through enzalutamide is effective in ERG expressing cells compared to ERG knockdown cells and that the expression of AR responsive genes were highly inhibited in ERG intact cells compared to knockdown cells.

### Enzalutamide inhibits bone tumor growth in TMPRSS2-ERG positive cells

**Fig. 3:**
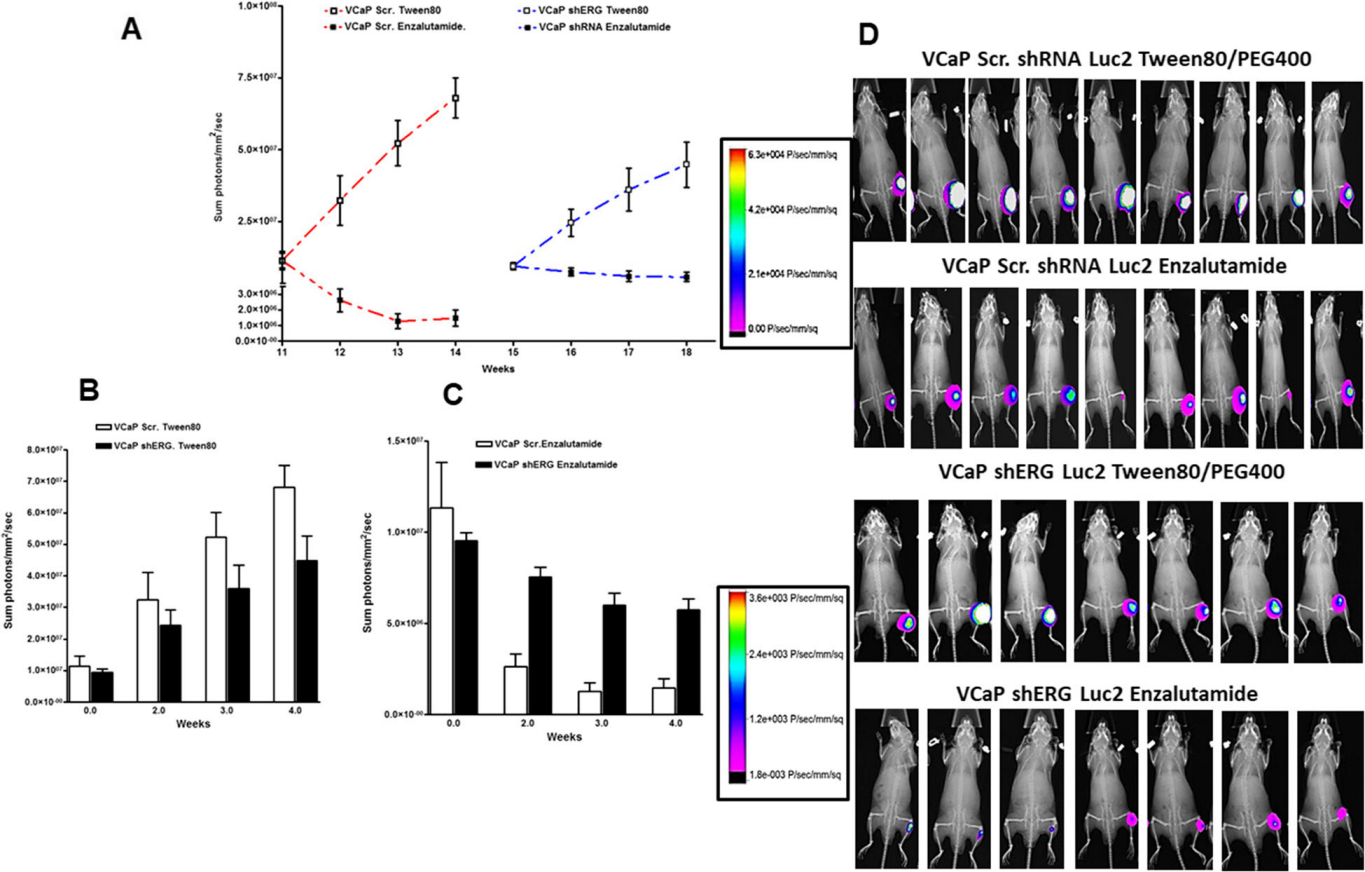
Enzalutamide inhibits the bone tumor growth. Enzalutamide treatment started when VCaP scr shRNA and VCaP ERG shRNA tumors reached similar size as per luciferase signal in the bone tumors. **a** Intratibial tumor growth of luciferase expressing VCaP scr shRNA and VCaP ERG shRNA cells and treatment with vehicle control or enzalutamide. **b** Comparison of tumor growth of vehicle treated VCaP scr shRNA and VCaP ERG shRNA tumors. **c** Comparison of tumor growth of enzalutamide treated VCaP scr shRNA and VCaP ERG shRNA tumors. **d** Luciferase imaging of bone tumors

Human bone tumor biopsy studies show that androgen production is present in bone tumors and activation of AR signaling contributes to bone tumor growth. To determine how ERG induced androgen synthesis contributes to bone tumor growth and whether ERG activity can be a predictor for anti-androgen therapy responsiveness, we utilized an intratibial bone tumor growth model with VCaP scr and ERG shRNA cells to address these questions. Tumor growth analysis of both cells lines show that VCaP ERG shRNA cells grow more slowly, requiring 4 weeks longer to reach similar growth sizes as assessed by luciferase imaging (Fig. 3a and d). When both tumors reach comparable size, the animals bearing bone tumors were randomized and treated with vehicle (Tween 80) or Enzalutamide by oral gavage. Tumor growth rate is slower in VCaP shERG tumors compared to scr shRNA tumors (Fig. 3b and d). Enzalutamide treatment resulted in significant reduction in tumor burden in VCaP scr shRNA group compared to VCaP shERG group (Fig. 3c and d). These data suggest that ERG signaling contributes to bone tumor growth and that AR targeting with enzalutamide significantly inhibits the bone tumor growth presumably through interfering with reduced ERG signaling.

### ERG knockdown and enzalutamide therapy inhibited androgen biosynthetic enzyme expression

**Fig. 4:**
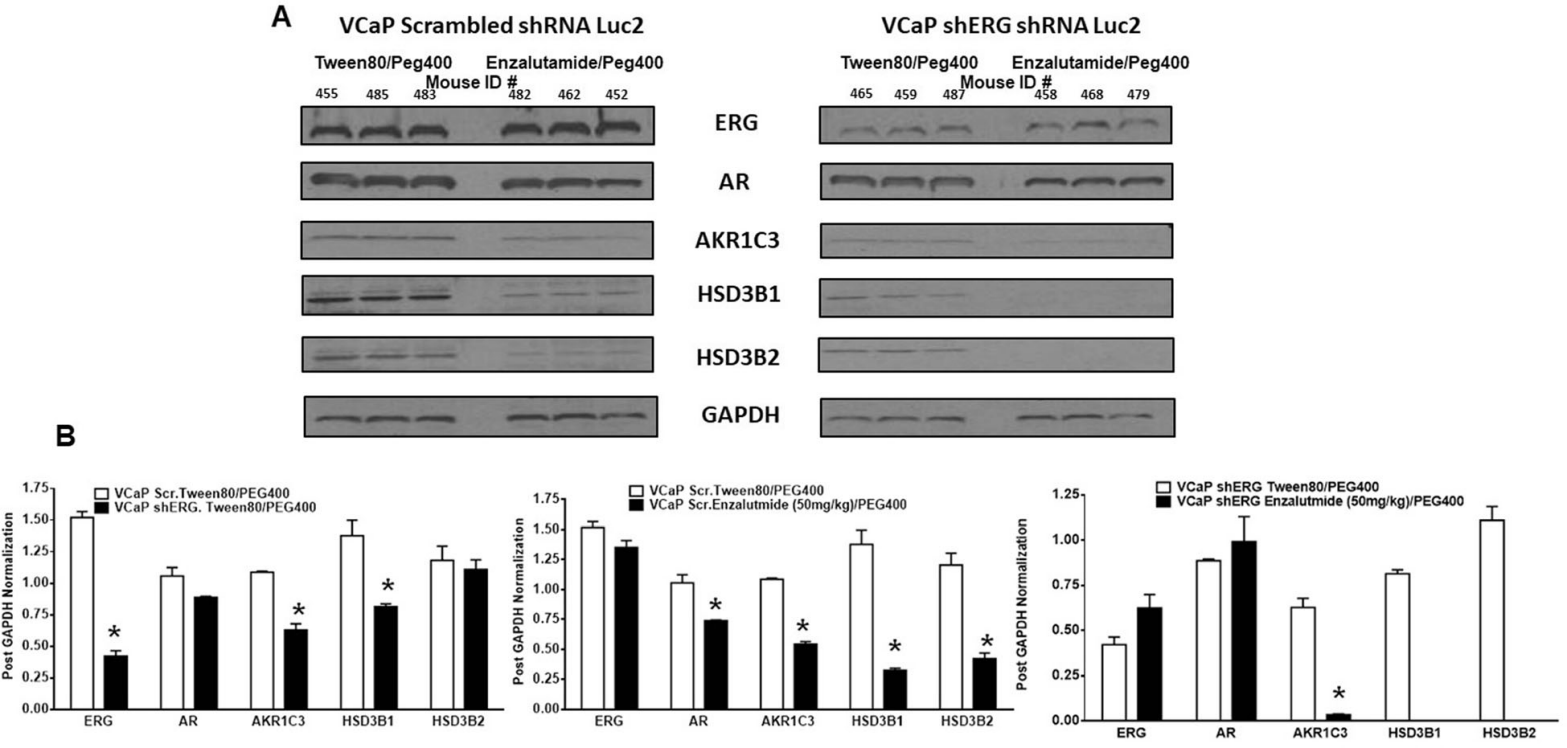
Enzalutamide inhibits androgen biosynthetic enzyme expression in bone tumors. **a** Westernblot analysis of VCaP scr shRNA and VCaP ERG shRNA tumors. **b** Quantitation of ERG, AR, AKR1C3, HSD3B1 and HSD3B2 in bone tumors

To determine enzalutamide responsiveness is related to androgen synthesis in bone tumors, we evaluated the expression of key enzymes in androgen synthesis, AKR1C3, HSD3B1 and HSD3B2, in bone tumors. Our previous study demonstrated that ERG factor regulates the expression of these enzymes in prostate cancer cells. Bone tumor analysis shows that ERG knockdown results in diminished expression of AKR1C3 and HSD3B1 in bone tumors (Fig. 4a and b). Enzalutamide treatment also inhibited the expression of all three enzymes in bone tumors. Intratumoral androgens in bone tumors were measured by MS/MS analysis. Testosterone was detected in vehicle treated tumors, whereas no detectable levels of testosterone were observed with enzalutamide treatment in both groups. This could be due to very small size of tumors which precludes the detectable limit of testosterone through MS analysis. The mean testosterone levels in VCaP scr shRNA tumors were 23.13 ng/mg protein whereas in VCaP shERG tumors, they were 8.73 ng/mg protein, suggesting ERG knockdown inhibited the testosterone production. These data suggest that ERG knockdown resulted in the reduced expression of androgen biosynthetic enzyme expression and corresponding lower levels of testosterone in bone tumors. Furthermore, enzalutamide therapy is more effective in ERG positive bone tumor at inhibiting tumor growth compared to ERG knockdown tumors.

## Discussion

Intratumoral androgen synthesis is the mainstay for AR signaling in castration resistance prostate cancer progression. Previous studies support this claim based on overexpression of several androgen biosynthetic enzyme expression coupled with enhanced utilization of adrenal precursors for DHT production in human CRPC tumors. To shed light on the transcriptional regulation of androgen biosynthetic enzymes in tumors, we have previously shown that ERG factor regulates key enzymes in androgen synthesis [11]; thus, TMPRSS2-ERG fusion positive cancer cells producing intracellular androgens are better responders to abiraterone therapy Herein, we extended this concept to in vivo tumor studies. We employed a model system of intact ERG expressing cells (VCaP scr ShRNA) and ERG knockdown cells (VCaP ERG shRNA) cells to test the role of ERG factor in anti-androgen receptor inhibitor (enzalutamide) responsiveness. Our data show that enzalutamide inhibited the growth of VCaP cells expressing TMPRSS2-ERG fusion genes, but when ERG factor was stably knocked down with lentiviral shERG RNA, tumor growth inhibition occurred at lesser extent. Previous study showed a similar enzalutamide dose (50 mg/kg body) inhibited subcutaneous LNCaP-AR tumors in the CRPC setting [28] suggesting that at this concentration the drug is stable and inducing the tumor regression, which is similar to its efficacy observed in clinical trials with the CRPC patients. Two recent clinical trials evaluated the efficacy of enzalutamide in prior chemotherapy [29] and chemotherapy naïve patients [30] with metastatic prostate cancer. The skeletal related events (SRE) such as number of bone lesions and bone pain was evaluated in these trials as a secondary endpoint. In the chemotherapy trial the enzalutamide significantly prolonged time to develop first SRE and in the chemotherapy naïve trial the enzalutamide significantly reduced the risk for developing first SRE in patients.

**Fig. 5:**
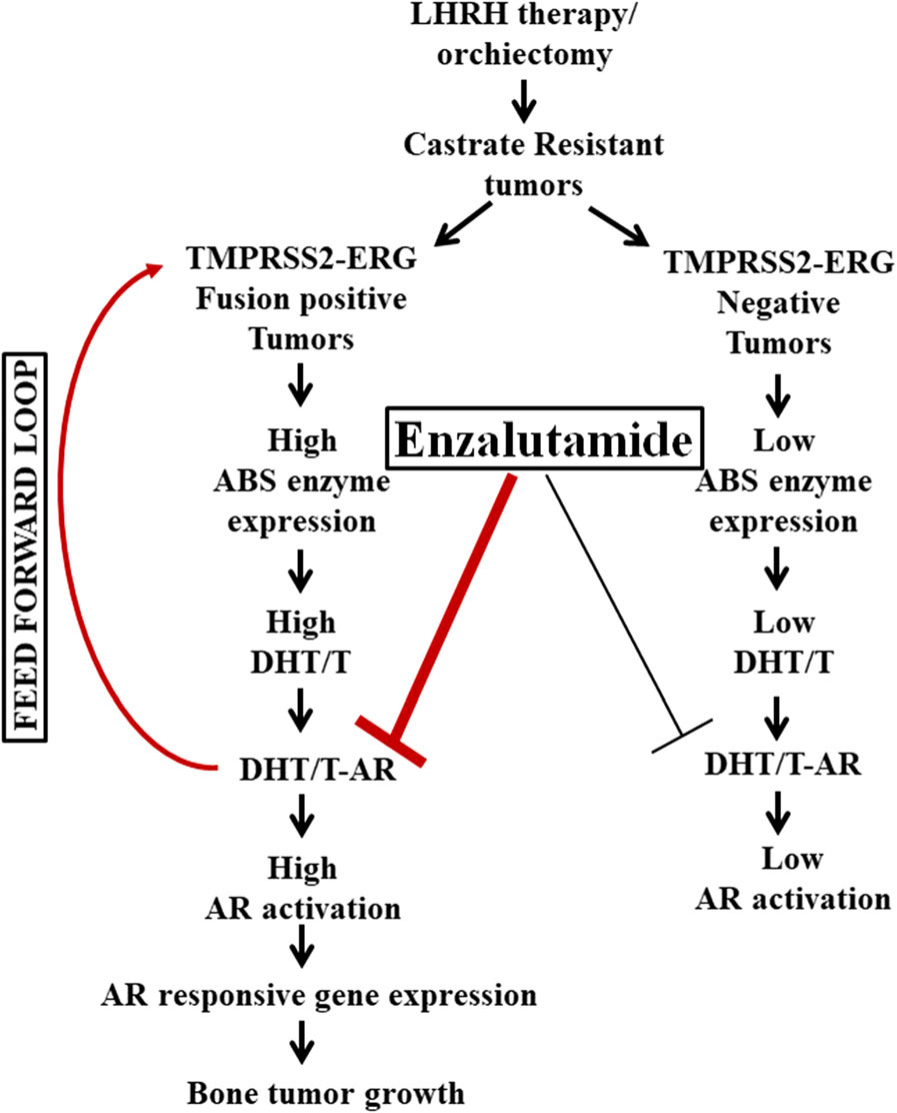
Enzalutamide is more potent in inhibiting the bone tumor growth of ERG expressing tumor cells

The data support the notion that ERG regulation of androgen synthesis in cancer cells leads to activation of AR as shown by AR responsive gene expression and this process is amenable to AR inhibition through enzalutamide for in vivo tumor growth, whereas when ERG factor is downregulated, intracellular androgen synthesis is downregulated and AR is no longer active (as shown by lower AR responsive gene expression), thus, not allowing anti-AR inhibitor enzalutamide to exert to growth inhibition to the extent of inhibition observed with ERG intact tumors (Fig. 5). Among androgen biosynthetic enzymes, we found that HSD3B1/2 and AKR1C3 and are key enzymes regulated by ERG factor in TMPRSS2-ERG fusion positive cells [11]. Enzymatic pathways of these two enzymes have been shown to be key determinants of DHT production through androstandione (5 a-dione) pathway, where HSD3B family enzymes catalyzes the irreversible step of converting DHEA to androstendione; and AKR1C3 is an aldo-keto reductase, which uses androstendione and androstanedione for testosterone and DHT production, respectively. Steroid receptor dehydrogenease 1, 2 converts testosterone to DHT as well as androstenedione to androstanedione, while previous study show that the for SRD1,2 enzymes androstendione is a preferred substrate over testosterone [31] thus overexpression of HSD3B1/2 and AKR1C3 in TMPRSS2-ERG fusion positive cells leads to production of DHT from adrenal androgens. Bone tumor analysis of these enzymes show that ERG knockdown tumors have lower expression, suggesting in vivo function of ERG in maintaining the enzyme expression in bone tumors. Enzalutamide treatment resulting in suppression of AKR1C3 and HSD3B1/2 in tumor cells leads to inhibition of bone tumor growth. Previous studies show that AKR1C3 has been upregulated in abiratarone resistant tumors [32, 33], suggesting that, in the AR responsive phase, androgen biosynthesis is inhibited but that, in resistance phase, tumor cells acquire the capacity to overexpress AKR1C3 enzyme. Further, treatment of abiraterone resistant tumors with AKR1C3 inhibitor indomethacin leads to inhibition of testosterone production in cells and in vivo tumor growth. Enzymatic function of AKR1C3 in maintaining DHT production in CRPC phase and overexpression of AKR1C3 upon anti-androgen therapy to restore the androgen production makes this enzyme a key component in the intracellular pathway operating in tumoral androgen biosynthesis. Furthermore transcriptional regulation by TMPRSS2-ERG in bone tumor growth reveals the reliance of bone tumor growth on intra-tumoral androgen synthesis.

HSD3B1 has been shown to be a predictive biomarker for developing the castration resistance phase of prostate cancer upon androgen ablation therapies. Genetic analysis reveals that 1245C germline variant encodes a gain-of function mutant of HSD3B1 which resulted in increased DHT production in tumors leading to the development of a castration resistance phenotype, and patients expressing this germline variant are more prone to develop CRPC [34]. Li et al., recently reported that HSD3B enzyme metabolizes the abiraterone to a more potent compound Delta (4)-abiraterone (D4A) which can further inhibit androgen biosynthetic enzyme activities involved in tumoral DHT production [35]. Our study also suggests that in ERG positive tumors this enzyme is overexpressed through a transcriptional mechanism for maintaining intra-osseous tumoral DHT production for expansion of tumors.

## Conclusions

Our data support the hypothesis that, in TMPRSS2-ERG fusion-positive cells, androgens are produced locally through transcriptional regulation of androgen biosynthetic enzymes. Thus, TMPRSS2-ERG fusions can serve as predictive biomarkers for patients undergoing for anti-AR therapies. Our in vivo data with bone tumor models demonstrating that enzalutamide can inhibit bone tumor growth and androgen biosynthetic enzyme expression in tumors, further suggest the effectiveness of anti-AR therapies for metastatic bone tumors.

## Data Availability

The data sets generated and/or analyzed during the current study available from corresponding author on reasonable request. The majority of data generated from the study are included in this published article.

## Abbreviations

AKR1C3: Aldo-Keto Reductase Family 1 Member C3
AR: Androgen Receptor
ATCC: *American Type Culture Collection*
BSA: Bovine serum albumin
CCNA2: Cyclin A2
CDC20: Cell Division Cycle 20
CDK1: Cyclin Dependent Kinase 1
CO_2_: Carbon Dioxide
CRPC: *Castration*-resistant prostate cancer
CYP17: Cytochrome P450 Family 17 Subfamily A Member 1
DHEA: Dehydroepiandrosterone
DHT: Dihydrotestosterone
DMEM: Dulbecco’s Modified Eagle Medium
ERG: ETS Transcription Factor
G_1_: Gap 1 phase
G_2_: Gap 2 phase
GAPDH: Glyceraldehyde-3-Phosphate Dehydrogenase
HEPES: (4-(2-hydroxyethyl)-1-piperazineethanesulfonic acid)
HPLC: High Performance Liquid Chromatography
HRP: *Horseradish peroxidase*
HSD3B1: Hydroxy-Delta-5-Steroid Dehydrogenase, 3 Beta- And Steroid Delta-Isomerase 1
HSD3B2: Hydroxy-Delta-5-Steroid Dehydrogenase, 3 Beta- And Steroid Delta-Isomerase 2
M: Mitosis
mM: *milliMolar*
MS: Mass spectrometry
NaF: Sodium fluoride
NaVO_3_: Sodium metavanadate
NIH: *National Institutes of Health*
PBS: Phosphate-buffered saline
PC: Prostate Cancer
PSA: Prostate-specific antigen
RIPA^++^: Radioimmunoprecipitation assay buffer with protease inhibitors
S: Synthesis phase
SCID: Severe combined immunodeficiency
Scr: Scramble
shRNA: short hairpin RNA
STR: Short Tandem Repeat
TBST: Tris-buffered saline, 0.1% Tween 20
TMPRSS2: Transmembrane Serine Protease 2
UBE2C: Ubiquitin Conjugating Enzyme E2 C
VCaP: Human Prostate; derived from metastatic site epithelial tissue
xg: *g*-*force* at accelerating
μg: microgram
